# Assessing the Relationship between Sense of Agency, the Bodily-Self and Stress: Four Virtual-Reality Experiments in Healthy Individuals

**DOI:** 10.3390/jcm9092931

**Published:** 2020-09-11

**Authors:** Yonatan Stern, Danny Koren, Renana Moebus, Gabriella Panishev, Roy Salomon

**Affiliations:** 1 Psychology Department, University of Haifa, Haifa 3498838, Israel; dkoren@psy.haifa.ac.il; 2 Gonda Brain Research Center, Bar-Ilan University, Ramat Gan 5290002, Israel; moebusrenana@gmail.com (R.M.); gabyp97@gmail.com (G.P.); roy.salomon@biu.ac.il (R.S.)

**Keywords:** sense of agency, metacognition, virtual reality, psychosis, stress, bodily-self

## Abstract

The bodily-self, our experience of being a body, arises from the interaction of several processes. For example, embodied Sense of Agency (SoA), the feeling of controlling our body’s actions, is a fundamental facet of the bodily-self. SoA is disturbed in psychosis, with stress promoting its inception. However, there is little knowledge regarding the relationship between SoA, stress, and other facets of the bodily-self. In four experiments manipulating embodied SoA using a virtual hand (VH), we examined (1) How is embodied SoA related to other facets of the bodily-self?; and (2) How is SoA impacted by stress? We found that increased alteration of the VH significantly decreased subjective ratings of SoA and body ownership (Exp. 1), supporting the close relation between SoA and body ownership. Interoceptive accuracy and SoA were positively correlated (Exp. 3), connecting awareness to one’s actions and cardiac signals. Contrary to our expectations, SoA was not related to trait anxiety (Exp. 3), nor did induced stress impair SoA (Exp. 4). Finally, we found a negative correlation between self-reported prodromal symptoms and SoA. These results strongly support the connection between SoA and the bodily-self. Whereas, SoA was not impaired by stress, and weakly related to psychotic symptoms.

## 1. Introduction

### 1.1. Sense of Agency and the Bodily-Self

Experiencing one’s self as a physical entity grounded in a body is termed the ‘bodily-self’ or ‘minimal self’, emphasizing the fundamental nature of this stratum of selfhood [1,2,3,4]. The bodily-self encompasses two related capacities: body ownership and sense of agency (SoA), which are typically seamlessly interwoven into our experience to such a degree that we are typically not explicitly aware of them, rather they are pre-reflective [5,6]. Body ownership, the identification with one’s body, relies on the integration of multisensory signals constructing a coherent representation of the self in the present moment. For example, synchronous visual–tactile stimulation can create an illusory sense of body ownership over a rubber hand or a virtual body [7,8,9]. SoA, the feeling of being in control of one’s actions, also relies on multisensory integration but incorporates internal neural efferent signals originating in the agent’s volition [10,11,12].

SoA primarily relies on the comparison of the predicted consequences of our actions (i.e., forwards models) with the actual afferent sensory outcomes via a comparator mechanism (but see other approaches such as Cue Integration, e.g., [13,14,15] and Postdictive accounts of SoA, e.g., [16,17]). If the predicted and actual outcome of the action match, then SoA arises, whereas if they are incongruent, the outcome will likely be attributed to an external cause [18,19,20]. For example, while reading this article on a computer screen, when you scroll the mouse with your finger to continue reading, and the screen scrolls, an SoA arises for these actions and outcomes. Importantly, two forms of SoA can be discerned in this example [21,22]. The first, embodied SoA, focuses on the immediate, often pre-reflective, connection between the intention to perform the action and the action itself (i.e., moving your finger) [23]. This form of SoA is inherently tied to the body of the agent that performs the action. The second, non-embodied SoA, focuses on the link between the action performed by the agent (i.e., moving your finger) and the intended outcome (i.e., screen scrolling). This SoA is related to a more general sense of causation, linking an action to its predicted outcome that is often detached spatially and temporally from the agent’s body and action. Historically, there has been no clear distinction between these two forms of agency, which are often conflated in the literature [11,24,25,26,27,28,29]. However, we argue that embodied SoA, with its close affinity to the bodily-self, introduces unique characteristics. The importance of this distinction is intuitively demonstrated by the hypothetical reaction to the failure of each form of agency. Imagine the profound shock and fear you would experience if you intended to move your finger, and it did not move (i.e., embodied SoA). Whereas, if you scrolled your mouse and the screen failed to scroll (i.e., non-embodied SoA), you would likely dismiss it as a problem with your laptop. In line with this distinction, the exclusive focus of the current paper is embodied SoA.

In the literature, various experimental paradigms have been used to examine SoA, yet these diverging paradigms probe different aspects of agency that are often conflated (see [22] for an extensive discussion). Thus, some paradigms (e.g., [30,31]) use an additional actor within the experimental setting, and probe agentic authorship by asking participants whether they or the other actor performed the presented action. While other paradigms have examined the role of active as opposed to passive actions on SoA (e.g., [26,28,32,33,34]), examining how the agent’s volition and the availability of neural efferent signals shape agency. In line with the theoretical suggestion that agency arises primarily from the congruence of predicted and actual sensory outcomes of action ([35,36,37]), and in line with a well-established line of research examining the effects of sensorimotor congruence on agency (e.g., [28,34,38,39,40,41]), the focus of the current paper is on examining how embodied agency is affected by the introduction of conflicts between actions and their visual consequences

### 1.2. Embodied SoA and Its Metacognition: Exteroceptive and Interoceptive Contributions

In line with our theoretical definition of embodied SoA that focuses on intention and motor actions, experimental operationalizations of embodied SoA use naturalistic representations of the body and focus on the motor action itself. Despite its theoretical and philosophical importance, experimentally examining embodied SoA has been somewhat limited, requiring creativity and ingenuity, such as using mirror set-ups and video systems [31,34], to create realistic distortions of self-generated movements. Recent advances in virtual reality (VR) technology allow for easier, more complex, and better-controlled manipulations of the bodily-self [9,42,43] and embodied SoA [28,44,45,46,47,48]. Such research has enabled significant advances in understanding the cognitive and neural processes by which sensory and motor signals shape the self [1,49,50]. In addition to the integration of exteroceptive signals, research has highlighted the importance of cardiac signals in the formation of the bodily-self. The neural processing of cardiac signals is modified by experimental changes in body ownership [51,52]. Moreover, cardiac signals can be used to induce body ownership over virtual hands [53] and bodies [54]. Within the realm of SoA, cardiac signals have been related to the subjective awareness of motor actions and errors [55,56]. Given the role of cardiac signals in body ownership and actions, we posited that awareness of one’s cardiac activity might be linked to awareness of one’s actions and embodied SoA.

Within judgments of SoA, we can distinguish between first-order and second-order processes [46,57,58]. First-order processes are related to the initial judgment of agency and include sensitivity—the ability to detect sensorimotor conflicts, and criteria—the decision threshold for asserting agency. Whereas, second-order processes concern the assessment of one’s initial judgment and include measures of confidence and metacognition. Importantly, metacognition is a bridge between immediate judgments of agency and reflective awareness and self-knowledge of one’s agency [5,57]. Both processes are central in delineating the self and are impaired in psychiatric populations such as psychosis [20,57,58]. Recently, we found that sensitivity and criteria of embodied SoA were both highly correlated across distortions in both the temporal and spatial domains introduced on a virtual hand presentation of self-generated movements [46]. These findings suggest an integrated mechanism of first-order processes that extends beyond specific domains and supports the processing of self-generated actions [4,10].

To briefly summarize, embodied SoA is a fundamental process contributing to our experience of the bodily-self. Accordingly, we hypothesize that SoA will be closely related to other facets of the bodily-self, such as body ownership and interoceptive processing. Furthermore, we expect that SoA and its metacognition will be correlated across domains, pointing to central processing and awareness of embodied actions.

### 1.3. Open Questions: The Effect of Stress

It is well documented that adverse situations such as stress and anxiety are a contributing factor to a wide range of psychopathological conditions entailing breakdowns of the self. Following trauma, people often experience dissociative states such as depersonalization that entail a deep sense of detachment from themselves, their body, and experiences [59,60,61,62]. Likewise, it is widely acknowledged that stress has a prominent role in the inception of psychosis [63,64,65]. Large-scale epidemiological studies have found that adverse life-events and extreme stress during childhood increase the odds of later psychotic experiences by 2–8 times [66,67]. Psychosis is characterized by disturbance of the minimal self, affecting the basic, pre-reflexive manner in which we experience ourselves, our body, and surroundings [3]. Accordingly, psychosis and disturbance of the bodily-self are closely linked to impaired SoA [68,69]. Across the psychosis continuum, impairments related to SoA are prominent both in the early prodromal phase [70,71] and in chronic schizophrenia patients [20,47]. Despite compelling evidence linking stress to psychosis and impairments of SoA, there is a paucity of experimental research examining how stress affects SoA. Experimentally understanding the effects of stress on SoA may allow us to uncover the underlying mechanisms and better understand the etiological factors affecting impaired SoA.

### 1.4. The Present Study: Goals and Predictions

The goal of the current study was to investigate two central questions using a VR paradigm of embodied SoA [46]. First, what is the relationship between embodied SoA and other facets of the bodily-self? Second, how is embodied SoA impacted by stress? These two questions were examined in a series of four experiments. Specifically, the following predictions were derived: (1) We hypothesized that increased sensorimotor conflict would significantly decrease subjective ratings of SoA and body ownership (Exp. 1). (2) We hypothesized that SoA processing (sensitivity, criterion and metacognition) would be correlated across different domains of sensorimotor conflict, suggesting a generalized mechanism for embodied SoA (Exp. 2). (3) In line with the role of cardiac signals in the formation of the bodily-self and motor awareness, we expected that interoceptive accuracy and embodied SoA would be positively correlated (Exp. 3). Concerning our second question, given the role of stress in the etiology of disturbances of the self, we expected that stress would be related to impaired SoA. Specifically, (4) we predicted that self-reported trait anxiety, in line with its close relation to stress [72], would be correlated with impaired SoA (Exp. 3). (5) Furthermore, we expected that experimentally manipulated psychosocial stress would affect SoA (Exp. 4). (6) Finally, in line with impairments of SoA in clinical populations with psychosis, we expected to find a correlation between a high presence of self-reported Attenuated Psychotic Symptoms (APS) in healthy individuals and impaired SoA (Exp. 2–4).

## 2. Participants

Participants in the study were 70 undergraduate students at Bar-Ilan University (37 women) aged 20–48 years (mean 25.4) All participants were right-handed, with normal or corrected-to-normal vision, and self-reported no history of neurological or psychiatric disease. They gave their informed consent to participate in the experiment and in return, were paid or given course credit. The study was approved by the Internal Review Board of Bar-Ilan University and was carried out in accordance with the relevant guidelines and regulations. Participants were excluded from the analysis if they did not have a sufficient number of trials (less than 80%), or failed to comply with instructions (see Supplemental Material Section A for details of participants excluded from each experiment).

## 3. Procedure

### 3.1. Experiment 1

**Figure 1:**
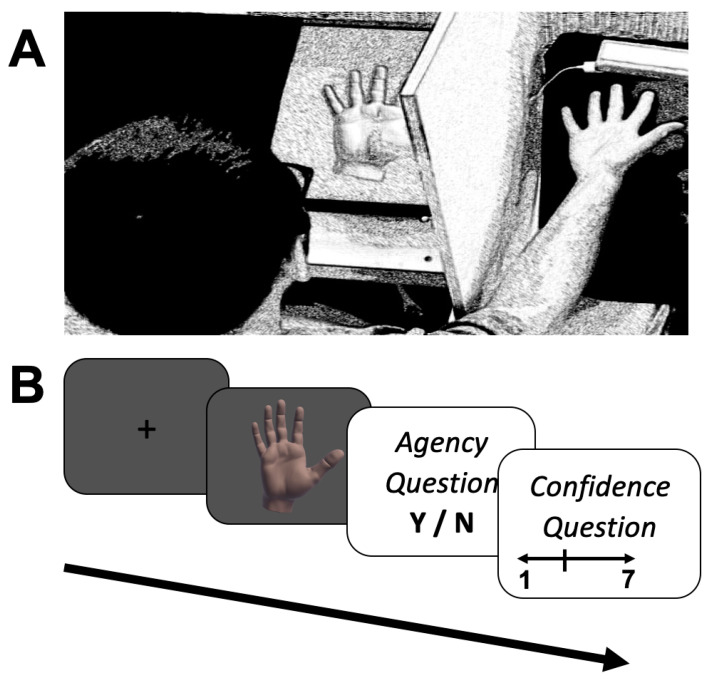
Experimental set-up and trial flow for the experiment. (**A**) Experimental set-up. The participant’s hand is hidden from his view by the barrier, and the camera mounted above his hand records movement and presents it on the screen in front of him as a VH. (**B**) Trial flow. In each trial, a fixation cross was followed by a VH that moved with/without an alteration. Following the VH’s presentation, in experiments 2–4, participants judged “same”/“not same” in response to the question “Was the movement of the VH identical to my movement?” (i.e., agency question) and rated their confidence in their response to the question, “How confident are you?” (i.e., confidence question) on a scale ranging from 1 (i.e., “not at all”) to 7 (i.e., “completely”). In experiment 1, participants rated their subjective experience of SoA (i.e., “I felt as if the movement presented was my own?”) and body ownership (i.e., “I felt as if the hand presented was my own?”) on a Likert scale ranging from −3 (i.e., “completely disagree”) to 3 (i.e., “completely agree”). Please note that across all experiments, the agency question included no explicit mention of the word ‘agency’.

In experiment 1 we examined whether increased magnitude of the sensorimotor conflict decreases subjective ratings of SoA and body ownership in order to validate that our experimental paradigm indeed assesses embodied SoA. Fourteen participants performed a VR-based paradigm of recognition of self-generated movements [46]. The technical set-up of the paradigm is described in detail in Krugwasser et al. (2019). In brief, participants’ right hand was placed 18 cm below a Leap Motion controller (Leap Motion Inc., San Francisco, CA, USA) and their hand was hidden from them via a barrier. A realistic Virtual Hand (VH) that mimicked the real hand’s position and movement was presented on a screen placed in front of the participant, positioned approximately 30 cm from the participant and at the same angle that the real hand was placed behind the barrier (see Figure 1A for set-up). In each trial, participants were presented with a fixation cross (1.5 s) followed by a presentation of the VH (2 s), during which they were instructed to fold only their index finger, while the rest of their hand remained static. A sensorimotor conflict was introduced in some trials by presenting the VH’s finger’s movement with one of four magnitudes of temporal delay (i.e., 0/100/200/300 ms) between the actual movement and the presentation of the VH’s movement. After the presentation of the VH, participants rated their subjective SoA (i.e., “I felt as if the movement presented was my own?”; questions were presented in Hebrew) and body ownership (i.e., “I felt as if the hand presented was my own”) on a Likert scale ranging from −3 (i.e., “completely disagree”) to 3 (i.e., “completely agree”) that were adapted from similar studies [26,28,73,74]. The order of the questions was counterbalanced across participants to avoid confusion. Thirty trials of each magnitude were presented, resulting in a total of 120 trials. A practice block consisting of 15 trials was performed with the experimenter present in order to ensure compliance with task demands at the start of the experiment and was not included in analyses. Trials were not analyzed if a camera malfunction occurred, as reported in the camera logs, if the participant did not respond to one of the questions, or if the reaction time exceeded four seconds.

### 3.2. Experiment 2

In experiment 2 we examined whether SoA performance across different sensorimotor domains is correlated, supporting a domain-general mechanism for embodied SoA. Nineteen participants performed the VH paradigm, with three key differences from experiment 1. First, in some trials, the VH was presented with an alteration either in the temporal or spatial domain and in other trials without an alteration. Importantly, alteration in both domains was not presented together in any of the trials. In the temporal domain, as in experiment 1, one of four magnitudes of delay (i.e., 0/100/200/300 ms) between the actual movement and the VH’s was inserted. In the spatial domain, an angular deviation of the VH’s movement was introduced, with four magnitudes of angular deviation (0°, 6°, 10°, 14°) inserted in the presentation of the bending movement of the index finger. Thus, during a 6° deviation trial the movement of the VH’s index finger would diverge in a lateral trajectory (i.e., towards the thumb) from the real index finger’s movement by 6°. The finger’s motion kinematics were modeled to ensure a natural-looking motion (see [46] for full details). Second, after the presentation of the VH, participants judged whether the VH’s movement was identical to their actual movement using a two-alternative forced-choice question (2 AFC), “Was the movement of the VH identical to my movement?” (i.e., agency question), and rated their confidence in their response (i.e., confidence question; see Figure 1B for trial flow). We used this phrasing for the agency question as it probes the psychophysical aspect of SoA (i.e., the sensorimotor correspondence), and is in line with previous studies that have involved altered feedback of self-generated movements [46,75,76,77], although other approaches using 3 AFC have also been employed (e.g., [24]). Third, participants performed in each domain, 40 trials of the zero (i.e., no alteration) and first magnitude, and 20 trials of the final two magnitudes. Thus, a total of 240 trials in random order were presented.

From this task, we calculated four measures pertaining to SoA and its metacognition. First, we calculated SoA as the proportion of trials in each magnitude of alteration in each domain that participants responded that the VH’s movement was identical to their movement. Second, we assessed confidence in SoA by averaging the confidence ratings of the trials with correct SoA (i.e., participants responded that the movement was identical when no alteration was present, or that it was different when an alteration was present). This was extracted for each magnitude of the alteration in each domain. Third, to allow for comparison of performance between domains, a signal detection framework was used to calculate sensitivity and criteria for each domain across the different magnitudes of alteration. Sensitivity reflects the capacity to accurately recognize trials in which the VH’s movement was identical to the real hand and trials that it was altered. Criterion reflects the individual’s decision boundary in order to judge that the VH movement was identical [78]. Finally, we calculated metacognitive confidence. In contrast to the measure of confidence described above, metacognitive confidence examines whether trials that were correct were rated with higher confidence than trials that were incorrect. This was calculated as the within-participant Goodman–Kruskal gamma correlation [79] between ratings of confidence and correctness of answers, capturing the degree to which confidence was linked to performance [80]. To ensure a sufficient number of observations, metacognitive confidence was calculated across the different magnitudes in each domain, yielding a single value per domain.

Following the completion of the task, participants completed the Prodromal Questionnaire Brief version (PQ-B) [81], as a self-report measure of APS that constitutes mild psychotic symptoms that have been experienced in the past month. We examined the number of items endorsed.

### 3.3. Experiment 3

In experiment 3 we examined the relation between SoA, interoception and self-reported anxiety. 23 participants performed the VH task identical to the task performed in experiment 2, except for two differences. First, in this experiment, only a temporal delay was administered. Second, 80 trials of each magnitude were administered in random order, resulting in a total of 320 trials. Following the VH task, participants performed a heartbeat tracking task. During the task, they were presented with a fixation cross for three time periods (25, 35, 45 s), in random order. They were instructed to press a button each time they perceived a heartbeat. During this period, the heart rate was recorded. Interoceptive accuracy was calculated as: $\frac{1}{3}\times \sum^{\;}1-\frac{\left|recorded\;heartbeats-counted\;heartbeats\right|}{recorded\;heartbeats}$ [82,83,84,85]. Accordingly, interoceptive accuracy values range from one, signifying perfect accuracy, to zero. Following the completion of the VH and heartbeat tracking task, they completed both the PQ-B, as in experiment 2, and the State-Trait Anxiety Inventory (STAI) [86]. The ratings of the STAI items were averaged, resulting in a score ranging from 0, reflecting low anxiety, to 4, reflecting high anxiety.

### 3.4. Experiment 4

In experiment 4 we examined the effects of experimentally induced stress on SoA. Fourteen participants performed a VH task similar to that in experiment 2 except for two differences. First, the VH task was split into two blocks, a ‘neutral’ block succeeded by a ‘stress’ block. Second, only the temporal domain was administered. For each magnitude of delay in each of the two blocks, 30 trials were presented, resulting in a total of 240 trials displayed in random order.

To experimentally induce stress, we used an adaptation of well-accepted stress manipulations such as the Trier Social Stress Test [87] and the Montreal Imaging Stress Task [88] that use peer-evaluation and assessment of performance as stressors. Specifically, at the start of the neutral block following the actual practice session with the experimenter, participants were informed that they would now perform a ‘practice’ block alone that is aimed at acclimating them to the task and that their performance will not be monitored. Following the ‘practice’ (i.e., neutral) block, the experimenter re-entered the room and briefed the participants that they will now perform the task as a test, and their performance will be monitored. To increase psychosocial stress associated with performing the task as a test, participants were briefed about a fictitious concept of ‘physical intelligence’, described in such a manner that it is a positive concept related to their self-esteem, and informed that the VH task is a measure of ‘physical intelligence’. They were informed that their performance in the current block will be compared to other participants’, and that high performers would be recruited for future studies that include high monetary reimbursement (see Supplemental Material Section B for description provided to participants). To further exacerbate social stress, a camera was set up opposite their face and they were informed that their facial expressions would be analyzed during this block, (in effect the camera did not record). Finally, participants were informed that their heart rate would be recorded during this block.

To assess the physiological reaction to the stress induction, during both blocks, heart rate was recorded from three electrodes placed on participants’ torso and connected to a G-Nautilus EEG system (250 Hz sampling rate) [89], and heart rate variability (HRV) was calculated. Briefly stated, HRV is a measure of the variability in the duration of succeeding R-peaks and is a robust biomarker of the body’s response to stressors [90,91,92]. Under stress, HRV is typically decreased, whereas during relaxation HRV is relatively high. Physiological stress reactivity was calculated by subtracting the HRV of the neutral block from the stress block. Importantly, negative values of this difference indicated increased stress during the stress block.

Following the completion of both blocks, participants completed a post-experiment interview with the experimenter in which they rated their subjective feeling of stress in the neutral and stress blocks on a Likert scale ranging from 1 (i.e., “not at all”) to 5 (i.e., “completely”). Subjective experience of stress was calculated by subtracting the neutral block’s score from the stress block’s score. In addition, the credibility of the stress induction was assessed in the interview. Finally, participants completed the PQ-B questionnaire.

Importantly, because this task will also be administered to clinical populations such as individuals at ‘clinical high-risk’ to develop psychosis in the future, we chose a stress manipulation that did not include negative feedback and could be successfully applied to them. Previous findings highlight that this population perceives even mildly stressful situations (i.e., solving simple mathematical problems with feedback introduced as a ‘neutral’ block) as extremely stressful [93], essentially equating them with the ‘stress’ block. Furthermore, based on our previous experience with clinical high-risk individuals in experimental settings in which participants were given negative feedback, we sought a mild stress manipulation that would ensure that they would complete the experiment and not leave due to the feedback.

## 4. Data Analysis

Data were processed using in-house Matlab scripts [94]. Statistical analyses were performed using JASP 0.9 [95]. To assess the effects of different magnitudes of alteration, domains, and blocks on subjective ratings of SoA and body ownership (Exp. 1), as well as SoA accuracy and confidence (Exp. 2–4), a within-subject repeated-measures ANOVA was used. In cases where the assumption of sphericity was violated, we performed Greenhouse–Geisser corrections. Post-hoc comparisons were Bonferroni corrected. To examine the correlations between variables, Pearson correlations were calculated. Finally, Bayesian statistics were used to assess evidence of null results [96,97].

## 5. Results

### 5.1. Experiment 1

**Figure 2:**
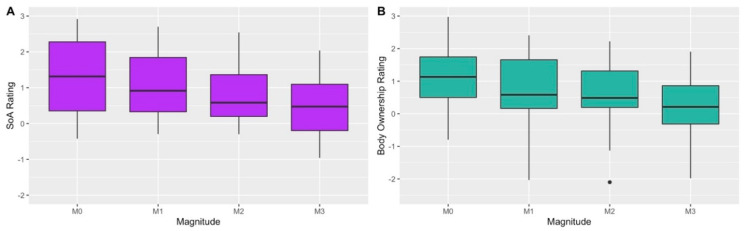
Subjective ratings of SoA (i.e., “I felt as if the movement presented was my own?”; Panel (**A**)) and body ownership (i.e., “I felt as if the hand presented was my own?”; Panel (**B**)) in experiment 1. *X*-axis is the magnitude of temporal alteration; *Y*-axis is the subjective rating that ranged from −3 (i.e., “completely disagree”) to 3 (i.e., “completely agree”).

To examine the effects of magnitude of alteration on subjective ratings of SoA and body ownership, two one-way repeated-measures ANOVAs with the factor magnitude (0/1/2/3) were performed. For SoA we found a significant main effect of magnitude (*F*_3,39_ = 6.72, *p* = 0.01, η^2^_p_ = 0.34; see Figure 2A), such that as magnitude of alteration increased ratings of SoA decreased. For body ownership we also found a significant main effect (*F*_3,39_ = 7.71, *p* = 0.006, η^2^_p_ = 0.37; see Figure 2B) similar to that of SoA. Thus, increased alteration of the VH’s movement decreased the subjective experience of both SoA and body ownership. To assess if SoA and body ownership were indeed experienced by the participants in the no alteration condition (i.e., M0), we performed a one-sample t-test examining whether M0 ratings significantly differed from the baseline rating of zero. Both SoA (*t*_13_ = 4.13, *p* = 0.001 Cohen’s *d* = 1.1) and body ownership (*t*_13_ = 4.13, *p* = 0.001, Cohen’s *d* = 1.1) were significantly greater than baseline, further strengthening the ecological validity of the VH paradigm. Finally, to compare the effect of alteration magnitude on SoA as opposed to body ownership, a 2 × 4 repeated-measures ANOVA with the factors magnitude (0/1/2/3) and embodiment aspect (SoA/body ownership) was performed. As expected, the effect of magnitude was significant (*F*_3,39_ = 8.03, *p* = 0.006, η^2^_p_ = 0.38); however, neither embodiment aspect (*F*_1,13_ = 1.29, *p* = 0.28), nor its interaction with magnitude (*F*_3,13_ = 1.36, *p* = 0.27) were significant. To assess this null finding, we examined the contribution of the embodiment aspect and its interaction with magnitude via the Bayes factor of inclusion (*BF_Inclusion_*). Briefly stated, *BF_Inclusion_* is a measure of the evidence supporting the inclusion of a given factor, by comparing the effect of including or stripping a given factor from a group of models with an equivalent number of parameters [98]. This analysis yielded inconclusive evidence for the inclusion of the embodiment aspect (*BF_Inclusion_* = 2.7), and moderately supported not including the interaction (*BF_Inclusion_* = 0.11). To further elucidate the relation between SoA and body ownership, for each participant we examined the correlation between the two ratings on each question. For all participants, the correlation was positive and significant (all *p*’s < 0.005) and the mean correlation across participants was 0.65 (SD = 0.25), with a range of 0.26 to 0.98. Thus, these two facets of the bodily-self are strongly entwined, and both were strongly affected by magnitude of alteration.

### 5.2. Experiment 2

**Figure 3:**
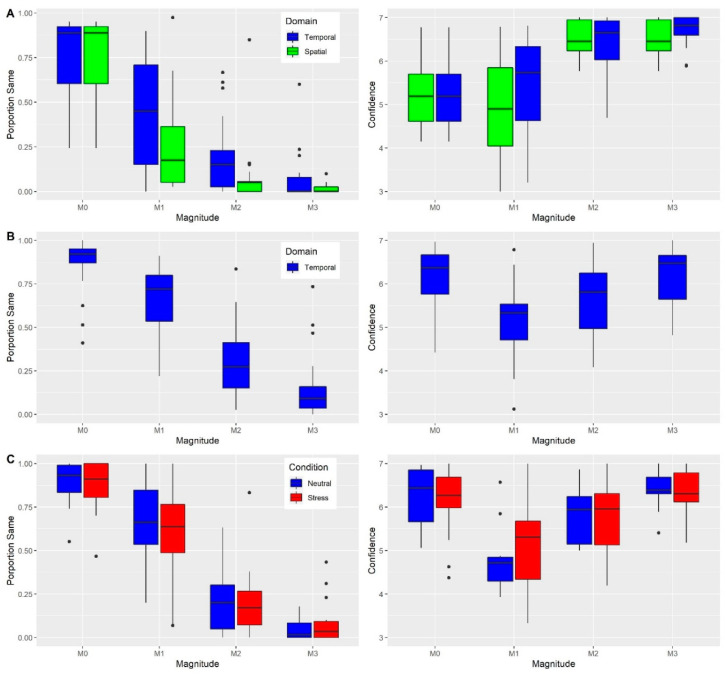
Judgments of SoA (left panels) and confidence (right panels) across three experiments (2–4; **A**–**C**). In the left panels, the *Y*-axis is the proportion of trials in which participants responded that VH’s movement was the same as the actual movement, across the different magnitudes of alteration. Importantly, only in M0 (i.e., no alteration) was it correct to respond ‘same’. In the right panels, the *Y*-axis is the participants’ mean confidence rating (rated on a scale of 1–7) of their response to the agency question in which their response was correct. Lines within colored boxes are medians. (**A**) Experiment 2. Alterations were introduced in both the spatial (green) and temporal (blue) domain. (**B**) Experiment 3. Alterations were only introduced in the temporal domain. (**C**) Experiment 4. Alterations were introduced only in the temporal domain, and assessed in a neutral (blue) and stress (red) block.

To examine the effects of domain and magnitude of alteration on SoA, a 2 × 4 repeated-measures ANOVA with the factor domain (temporal/spatial) and magnitude (0/1/2/3) was used. A significant main effect of domain was found (*F*_1,54_ = 8.87, *p* = 0.008, η^2^_p_ = 0.33; see Figure 3A), such that in the temporal domain participants tended to judge VH movements to be identical to their own more than in the spatial domain. Importantly due to the distribution of trials across the magnitude of alteration, this main effect reflects participants’ increased tendency to mistake altered trials in the temporal domain. In light of this main effect of domain, in order to examine the effect of magnitude on SoA, we performed a one-way repeated-measures ANOVA with the factor magnitude (0/1/2/3) for each domain separately. In the temporal domain we found a significant main effect of magnitude (*F*_3,54_ = 57.44, *p* < 0.001, η^2^_p_ = 0.76; see Table S1 in the Supplemental Material for summary statistics and pairwise comparisons), as well as in the spatial domain (*F*_3,54_ = 78.35, *p* < 0.001, η^2^_p_ = 0.81). As expected, in both domains, as the magnitude of alteration increased, VH movements were correctly judged as not identical to the participants’ actual movements. Finally, there was a significant interaction between magnitude and domain (*F*_3,54_ = 6.41, *p* < 0.001, η^2^_p_ = 0.26). As can be seen in Figure 3A, the differences between domains were most prominent in magnitude one and two, with participants showing a greater tendency to misattribute movements in the temporal domain in these magnitudes.

An equivalent repeated-measures ANOVA was used to examine the effects of domain and magnitude of alteration on the confidence of correct trials. A significant main effect of domain was revealed (*F*_1,51_ = 6.64, *p* = 0.02, η^2^_p_ = 0.27; see Figure 3A), such that participants were more confident in the spatial domain. This finding complements our previous findings that participants were more accurate in the spatial domain. In light of this main effect of domain, in order to examine the effect of magnitude on confidence, we performed a one-way repeated-measures ANOVA with the factor alteration magnitude (0/1/2/3) for each domain separately. In the temporal domain we found a significant main effect of magnitude (*F*_3,51_ = 13.89, *p* < 0.001, η^2^_p_ = 0.45; see Table S2 in Supplemental Material for summary statistics and pairwise comparisons), as well as in the spatial domain (*F*_3,54_ = 25.77, *p* < 0.001, η^2^_p_ = 0.60). Contrary to our expectations and findings in subsequent experiments, in the no alteration magnitude, confidence ratings were relatively low. This may be due to the high number of trials with alterations of magnitude zero (i.e., no alteration) and one, that caused participants to be uncertain whether a slight alteration or no alteration occurred.

To further elucidate the relation between SoA in the different domains, we examined the between-subject correlation of sensitivity, criterion and metacognitive confidence between the temporal and spatial domains. For sensitivity, we found a strong correlation between domains (*r* = 0.71, *p* < 0.001). Likewise, for criterion, there was a strong correlation between domains (*r* = 0.89, *p* < 0.001), reflecting that the threshold for evidence used for judgments of agency were related across domains. Finally, for metacognitive confidence we also found a strong correlation between domains (*r* = 0.83, *p* < 0.001). These findings demonstrate that the mechanism underlying embodied SoA and its metacognition are not domain-specific rather rely on a domain-general mechanism perhaps related to the bodily-self. Importantly, these findings are a strong replication of our previous findings concerning across domain correlations for sensitivity and bias [46], and also extend them to the capacity of metacognitive confidence.

Finally, we examined the relation between SoA and APS, assessed via the PQ-B questionnaire. We found a significant negative correlation between APS and sensitivity in the spatial domain (*r* = −0.68, *p* = 0.001) and in the temporal domain (*r* = −0.49, *p* = 0.03). Thus, participants that reported more APS symptoms showed lower sensitivity in both domains. Furthermore, the relation to metacognitive confidence, in both the spatial (*r* = −0.65, *p* = 0.003) and the temporal (*r* = −0.57, *p* = 0.01) domains was significant. Thus, participants that reported higher APS had lower metacognitive confidence.

### 5.3. Experiment 3

To examine the effect of the magnitude of alteration on SoA a one-way repeated-measures ANOVA with the factor of magnitude (0/1/2/3) was applied. Replicating our finding in experiment 2, a significant main effect of magnitude was found (*F*_3,66_ = 121.3, *p* < 0.001, η^2^_p_ = 0.85). As the magnitude of alteration increased the proportion of trials judged to be identical decreased (see Figure 3B). Examining the effects of magnitude on the confidence of correct trials, using an equivalent ANOVA, also revealed a significant effect of magnitude (*F*_3,66_ = 15.33, *p* < 0.001, η^2^_p_ = 0.41). As can be seen in Figure 3B, a U-shaped curve of confidence was observed, with the lowest confidence exhibited in magnitude one of alteration. Presumably, this magnitude of alteration is the most difficult, in line with its high proportions of incorrect responses. As expected, and in contrast to experiment 2, the no alteration condition (i.e., magnitude 0) also exhibited high levels of confidence. Presumably, this reflects the relative ease of correctly identifying movements that are identical.

Examining the correlation between interoceptive accuracy, assessed via the heartbeat counting task, and sensitivity of SoA, assessed via the VH task, we found a significant positive correlation (*r* = 0.52, *p* = 0.02). Similarly, the correlation to metacognitive confidence was also significantly correlated (*r* = 0.47, *p* = 0.04). Thus, individual differences in interoceptive accuracy and both sensitivity and metacognition of embodied SoA were closely linked, as would be expected due to these capacities’ close affinity to the bodily-self.

Concerning the relation between the self-reported measures of APS and trait anxiety, and the performance measures of SoA and interoceptive accuracy. Contrary to our hypothesis and the results of experiment 2, the correlation between sensitivity of SoA and APS was not significant (*r* = −0.05, *p* = 0.81), with Bayesian statistics providing anecdotal evidence for lack of a correlation (BF_01_ = 2.97). Neither was the correlation between metacognitive confidence and APS significant (*r* = −0.17, *p* = 0.45), with Bayesian statistics providing anecdotal evidence for a lack of correlation (BF_01_ = 2.94). Concerning trait anxiety, the sensitivity of SoA was not significantly correlated (*r* = −0.15, *p* = 0.52), with Bayesian statistics providing moderate evidence in favor of a lack of correlation (BF_01_ = 3.1). Meanwhile, interoceptive accuracy and anxiety showed a non-significant negative correlation (*r* = −0.36, *p* = 0.13), such that participants with higher levels of anxiety had lower interoceptive accuracy.

### 5.4. Experiment 4

In this experiment, we examined the effects of experimentally manipulated stress on SoA. First, we assessed the validity of the stress induction both via the subjective experience of stress as reported in the post-experiment interview in which participants rated on a Likert scale ranging from 1–5 how stressful they experienced each block to be, and via the physiological change in HRV between blocks. As expected, the mean rating of stress experienced in the stress block (M = 2.68, SEM = 0.24) was higher than in the neutral block (M = 2.00, SEM = 0.22), and this difference was significant (*t*_18_ = 3.24, *p* = 0.005, Cohen’s *d* = 0.74). In contrast, the difference between HRV in the stress block (M = 0.049, SEM = 0.005) and the neutral block (M = 0.052, SEM = 0.005) was not significant (*t*_13_ = 0.49, *p* = 0.63). Importantly, the subjective experience of stress was correlated with the change in HRV (*r* = −0.64, *p* = 0.014). Thus, changes in HRV reflect individual differences in the subjective experience of stress following its induction. Accordingly, in the analyses concerning the effects of stress, we included HRV as a covariate to capture these individual differences.

We examined the effect of stress on SoA via a repeated-measures ANOVA with the factors block (neutral/stress) and magnitude of alteration (0/1/2/3), and change in HRV as a covariate. There was a significant main effect of magnitude (*F*_3,36_ = 86.71, *p* < 0.001, η^2^ = 0.87; see Figure 3C), replicating our previous findings concerning the decrease in SoA as magnitude increases. The main effect of block was not significant (*F*_1,12_ < 1, *p* = 0.40). Meanwhile, the interaction between block and delay was significant (*F*_3,24_ = 3.56, *p* = 0.024, η^2^ = 0.20), with participants under stress more likely to misattribute their movement in the largest magnitude of alteration. Yet none of the post-hoc comparisons between blocks across the different magnitudes of alteration were significant (*p* > 0.12). Comparing sensitivity of SoA between blocks, contrary to our expectation the difference between sensitivity in the stress block (M = 1.78, SEM = 0.22) and the neutral block (M = 1.75, SEM = 0.19), was not significant (*F*_1,12_ < 1, *p* = 0.37). The equivalent Bayesian analysis yielded anecdotal evidence in favor of the null effect of the block (BF_01_ = 2.67). Thus, it seems that the stress induction did not affect overall SoA.

Examining the effect of stress on confidence with the equivalent ANOVA used for SoA, we found a significant main effect of magnitude (*F*_3,24_ = 9.74, *p* < 0.001, η^2^ = 0.54; see Figure 3C). Replicating experiment 3, confidence across magnitudes displayed a U-shaped curve with the lowest confidence in the first magnitude of alteration. The main effect of block was not significant (*F*_1,8_ = 2.63, *p* = 0.14), nor was the interaction of block and magnitude (*F*_3,24_ = 1.1, *p* = 0.37). Comparing metacognitive confidence between blocks, under stress metacognitive confidence was higher (M = 0.43, SEM = 0.08) in comparison to the neutral block (M = 0.41, SEM = 0.07), and this difference was significant (*F*_1,12_ = 10.6, *p* = 0.007, η^2^_p_ = 0.47). Thus, contrary to our hypothesis, stress significantly improved metacognitive confidence.

Finally, examining the correlation between sensitivity of SoA and APS, contrary to our expectation, we did not find a significant correlation (*r* = −0.07, *p* = 0.79), with Bayesian analysis providing anecdotal to moderate evidence for lack of a correlation (BF_01_ = 2.98).

### 5.5. The Relation between APS and SoA across Experiments

**Figure 4:**
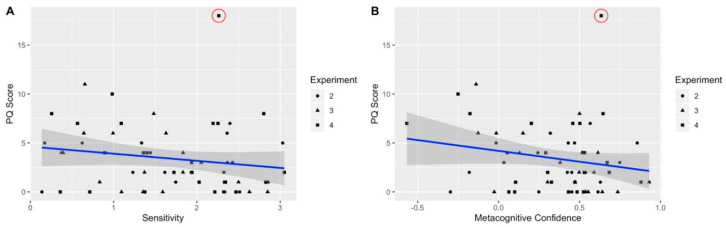
Correlation between APS and sensitivity (**A**) and metacognitive confidence (**B**) across the three experiments (experiment 2–4). The blue line denotes the linear regression fit and the shaded area is 95% confidence interval of fit. A red circle surrounds the participant identified as an outlier.

To further examine the relation between APS and SoA with increased statistical power, we pooled the participants from the three experiments (i.e., Exp. 2–4) in which the binary judgment of SoA was used and sensitivity could be extracted, and examined only SoA in the temporal domain, because it was measured in all three studies (in experiment 4 we included only the neutral block). APS showed a negative correlation to sensitivity (*r* = −0.17, *p* = 0.19; see Figure 4A) and metacognition of SoA (*r* = −0.20, *p* = 0.12; see Figure 4B), yet contrary to our expectation these correlations were not significant. Noting that one participant in experiment 4 endorsed an extremely high number of APS symptoms (18 out of 21 items), we reexamined the correlation, excluding this participant as an outlier. There are two justifications for this decision. First, clinical cutoffs of the PQB are typically set at between 3 to 8 items [81,99]; thus the participant’s high number of items endorsed likely points to his misunderstanding of the questionnaire or extreme clinical distress that was not evident in his functioning. Second, the number of items endorsed by this participant is more than three standard deviations from the participants’ mean number of items endorsed. Excluding this participant, there was a significant correlation between APS and sensitivity (*r* = −0.27, *p* = 0.04) and metacognitive confidence (*r* = −0.32, *p* = 0.01) of SoA. Yet, it should be noted that these correlations were not corrected for multiple comparisons.

## 6. Discussion

Across four experiments, using a virtual reality paradigm, we examined two questions pertaining to embodied SoA. The first question focused on the relationship between embodied SoA and other facets of the bodily-self, such as body ownership and interoception. All of our predictions derived from this question were corroborated. First, we found that increased magnitude of alteration significantly decreased graded subjective ratings of both SoA and body ownership, validating the ecological validity of our experimental paradigm of embodied SoA (prediction1; experiment1). These two measures exhibited strong within-trial correlations, further supporting a unified construct of the bodily-self. This finding is in contrast to theoretical and empirical accounts [26,100] that argue for a dissociation between these two processes and is in line with previous studies that have found the two to be correlated [28,73]. Second, our prediction that sensitivity and criteria would be correlated across temporal and spatial domain (prediction 2; experiment 2) was strongly supported and replicates the findings of our previous study [46]. These correlations support an account of a domain-general processing of embodied SoA, differing between individuals but stable across different sensorimotor prediction domains. The current study also expanded this correlation across domains to the capacity of metacognitive confidence of SoA. Metacognitive confidence, reflecting the degree to which the participant is aware of his performance, bridges between immediate judgments of agency and more reflexive knowledge pertaining to the certainty of these judgments [57]. Correlated metacognition together with correlated criterion point to similar subjective processes inherently related to embodied SoA, lending further support to the existence of a central, domain-general representation of the bodily-self. Future neuroimaging studies may attempt to examine whether embodied SoA across domains involves similar or distinct neural mechanisms and regions. In line with our third prediction and further supporting embodied SoA’s connection to the bodily-self [2,52,101,102,103], we found that interoceptive accuracy was significantly correlated with SoA (prediction 3; experiment 3). Previous studies have found that interoceptive accuracy is linked to body ownership [84] and peripersonal space [85]. The current finding demonstrates the importance of awareness to cardiac signals in influencing enactment, that alongside embodiment, shape our representation of the bodily-self.

A limitation pertaining to our finding that SoA was closely related to the bodily-self is that in the current study (Exp. 2–4) we only examined one aspect of SoA, namely the effect of sensorimotor alterations on SoA. Importantly, it should be noted that SoA entails additional aspects [22], such as agentic authorship (i.e., did I or someone else perform the action [24,31]) as well as the effect of passive as opposed to active actions and the effect of volition on SoA [29,104,105]. Furthermore, the wording of experiment 1’s question (i.e., “I felt as if the movement presented was my own”) is ambiguous in the sense that it may be interpreted as probing either the sensorimotor congruency or the agentic authorship aspect of SoA. Hence, additional studies are needed in order to disentangle the relationship of these additional aspects of SoA to the bodily-self. Additionally, Exp. 1 only probed the effect of alterations in the temporal domain on SoA. In light of previous findings that have found significant differences between domains and their effect on different aspects of SoA [24], future studies should also examine the effects of spatial alterations on the subjective experience of SoA.

Examining the relation between impaired SoA and APS yielded precarious results (prediction 6). Only in experiment 2 was the correlation to SoA in both domains significant, whereas in both experiments 3 and 4, we did not find significant correlations and Bayesian analyses provided anecdotal to moderate evidence for lack of a correlation. Pooling participants from all three experiments, the correlation was only significant when excluding an outlier. From these mixed findings, we derive two preliminary conclusions that although consistent with existing research, nonetheless need to be replicated in future studies. First, the significant correlations found between self-reported APS and embodied SoA are in line with the ‘psychosis continuum’ that posits the existence of psychotic-like experiences also in normative functioning populations [106]. Importantly, the current study expands previous studies [70] by showing that APS is related to both impaired sensitivity of embodied SoA and its metacognition. Interestingly, a recent study using VR to manipulate other aspects of the embodied Self (i.e., making the participant bigger or smaller) also showed a correlation between sensitivity to this alteration and APS [107]. Our correlation between self-reported psychotic symptoms and metacognition points to the potential role metacognition may play in attenuating and translating experimentally measured impairments into real-world functioning [108,109,110]. Second, the precariousness of the correlations highlights the wide range of symptoms endorsed in the general population, which especially affects small sample sizes. To clarify, we fully acknowledge the seminal importance of self-report questionnaires in the early stages of screening for risk of psychosis [81,99,111]. Yet, due to the low specificity of the questionnaires, some individuals endorse a high number of items that may not be indicative of actual APS and introduces additional noise to correlational analyses. Accordingly, we interpret these mixed findings with cautious enthusiasm and believe that they require further replication.

Our second question based on the pivotal role of stress in breakdowns of the self and psychosis examined the effects of stress on SoA. It yielded complex findings that did not corroborate our two predictions. The first prediction (prediction 4; experiment 3) concerning the correlation between trait anxiety and SoA was not corroborated. Although stress and anxiety are closely related concepts [72], a single self-report questionnaire of anxiety may not capture the ‘stress-sensitivity’ that has been found to be elevated during the early stages of psychosis. It is typically assessed at numerous time points over an extended period, via the experience sampling method that allows us to assess participants’ reaction to daily stressors [112,113,114]. Future studies will use this method, which captures minute fluctuations in both anxiety and subjective stress, as well as obtaining a more stable measure of stress reactivity over an extended period. We also note the small sample size and low statistical power may have impaired the detection of a potential relationship. Furthermore, contrary to our prediction, we found that experimentally manipulating mild psychosocial stress in healthy individuals improved metacognitive confidence and did not affect the sensitivity of SoA (prediction 5; experiment 4). In line with the well-established inverted U-shape effect of stress on performance [115], we hypothesize that the mild stress placed healthy participants in the upward part of the inverted U, as evidenced by their enhanced metacognition. This finding highlights the importance of using a graded manipulation of stress that will allow us to calibrate the effects of the stress manipulation on the participant’s subjective experience, allowing us to compare participants’ performance at an equated level of subjective stress.

Although stress did not impair SoA in the current experiments, we nonetheless believe that paradigms integrating virtual reality and stress inductions are a promising avenue. It is well recognized that preceding full-blown psychosis and schizophrenia, most individuals undergo a period of heightened APS, in a risk period often coined ‘clinical high-risk’ [116,117,118]. Improving our ability to accurately predict which at-risk individuals will eventually convert to full-blown schizophrenia is a pressing goal that holds a potential to improve the harsh prognosis of schizophrenia. This has led to an ongoing search for neurocognitive characteristics and biomarkers specific to the clinical high-risk population in general and convertors in particular [71,119,120]. Yet the correlation between various impairments and the actual prognosis is typically weak at best. In line with the prominent role of stress in the etiology of psychosis and at-risk individual’s heightened reactivity to mild daily stressors [112,113], we have recently hypothesized that neurocognitive performance under stress may be of greater predictive value than neurocognitive performance per se [108]. Analogous to cardiac patients that during rest exhibit an electrocardiogram similar to healthy controls and only under cardiac stress (i.e., walking on a treadmill) is their signal differentiable. Neurocognitive deficits during the at-risk phase may only be identifiable in a stressful environment that mimics the environment in which psychosis typically occurs [108,121]. Importantly, experiment 4 served as a pilot study to examine the effect of stress on neurocognitive capacities associated with the bodily-self in at-risk populations. In the hope that performance under stress will yield insight into the etiology of psychosis and improve our ability to predict conversion.

## 7. Conclusions

The current study examined embodied SoA using a virtual reality paradigm. We found a strong connection between embodied SoA and other facets of the bodily-self, both replicating our previous finding [46] that SoA is highly correlated across different domains of alteration, and finding that it is correlated with interoceptive accuracy and body ownership. In addition, embodied SoA was correlated with self-reported psychotic symptoms, further supporting the disturbance of the bodily-self in the psychosis continuum. Finally, examining how embodied SoA is impacted by stress in a normative population, we found that it was not impaired. Despite this finding, which was contrary to our hypothesis, we believe that examining the effects of stress on embodied SoA in clinical populations holds promise for better understanding the role of stress in the etiology of psychosis.

## Supplementary Materials

The following are available online at https://www.mdpi.com/2077-0383/9/9/2931/s1, Supplemental Material Section A: Exclusion Criteria and Number of Participants Excluded from Experiments, Supplemental Material Section B: Description of Brief included in the Stress Induction of Experiment 4, Supplemental Material Section C: Tables of Summary Statistics for VH task, Table S1. Summary statistics of the VH task across the four experiments, Mean (S.E.M), Table S2. Summary statistics of the confidence rating of correct responses in the VH task across the experiments 2–4, Mean (S.E.M).
