# Supplementary material for: Assessing the Relationship between Sense of Agency, the Bodily-Self and Stress: Four Virtual-Reality Experiments in Healthy Individuals
Source: J Clin Med. 2020 Sep 11;9(9):2931. doi: 10.3390/jcm9092931 (PMC7563244; doi:10.3390/jcm9092931)
Supplement: Supplementary file 1 [file jcm-09-02931-s001.pdf]

Supplemental Material Section A: Exclusion Criteria and Number of Participants Excluded from Experiments

Exclusion Criteria

There were two levels of exclusion, trial and participant.

Trials were excluded for the following reasons: (1) Participant answered agency question after more than 4 seconds. (2) The participant did not respond to the agency question. They are instructed not to respond if there is a Leap camera malfunction. (3). Camera malfunction was reported in Leap camera logs.

Participants were excluded for the following reasons: (1) They were missing more than 20% of all trials. In experiment 4, where heart rate was also measured during task performance, participants were also removed if more than 20% of heart rate recording was contaminated. Heart rate was manually reviewed by a researcher (R.M) that was blind to experimental conditions, and she removed segments that were contaminated by movement and electrical noise. (2) Participants showed negative sensitivity on VH task. (3) In experiment 4, participants were excluded if they did not believe the stress induction manipulation, as assessed in the post-experiment interview.

Number of Participants Excluded from Each Experiment

In experiment 1, 15 participants performed the experiment. One participant was excluded from the analysis because of an insufficient number of trials.

In experiment 2, 20 participants performed the experiment. One participant was excluded from the analysis because of an insufficient number of trials. In experiment 3, 26 participants performed the experiment. Two participants were excluded from the analysis because of an insufficient number of trials in the VH task. A third participant was excluded due to failure to comply with the heartbeat counting task, reporting of fewer than ten heartbeats per time interval, and an interoceptive accuracy score that was more than 3 standard deviations from the group mean. In experiment 4, 20 participants performed the experiment. Two participants were excluded from the analysis because they did not believe the stress manipulation as assessed in the post-experiment debriefing. The first six participants were also excluded due to the failure of ECG recording equipment that was fixed for the remaining participants.

Supplemental Material Section B: Description of Brief included in the Stress Induction of Experiment 4

To experimentally induce stress in experiment 4, at the start of the neutral block following the actual practice session with the experimenter, participants were informed that they would now perform alone a ‘practice’ block that is aimed at acclimating them to the task and that their performance will not be monitored. Following the ‘practice’ (i.e., neutral) block, the experimenter re-entered the room and briefed the participants that they will now perform the task as a test and their performance will be monitored. To increase psychosocial stress associated with performing the task as a test, participants were briefed about a fictitious concept of ‘physical intelligence’, described in such a manner that it is a positive concept related to their self-esteem. In addition, they were informed that their heart rate will be recorded. A camera was set up to record their facial expressions, and they were informed that the recording would be analyzed by expert behavioral analysts. The following is the translation (from Hebrew) of the document presented to the participants:

"<u>Background:</u> As you have probably experienced, a large portion of interpersonal communication does not occur via verbal communication, but rather through non-verbal communication and body language. In the past decade, studies have found that in addition to explicit (conscious) body language such as facial expressions, there is also an implicit (unconscious) body language that relies on rapid (faster than 500 msec) and minor changes in facial expressions, eye movement, perspiration, heart and respiratory rates. These changes are coined "micro-expressions", and they contribute greatly to the quality of interpersonal interactions. Furthermore, they often convey information that the person may be attempting to hide or is unaware of. According to theories of evolution, micro-expressions depend on activity in the amygdala, part of the limbic brain region. A primal brain region in charge of emotions that are crucial for survival, such as fear, anger, and intuitive, rapid decision-making processes (see Figure below).

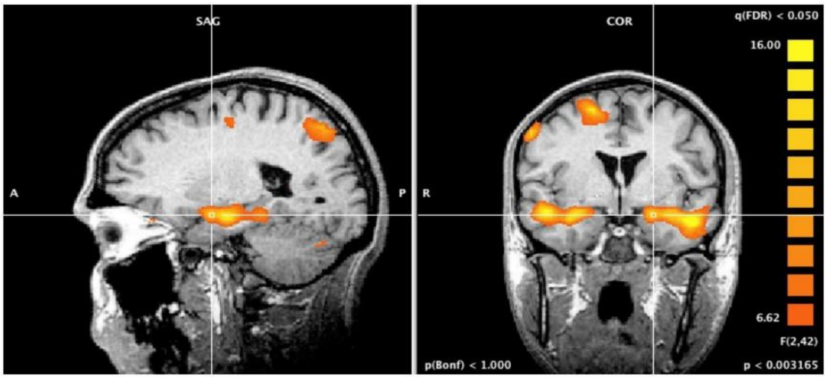

Brain activity in the amygdala while participants watch a scary movie

Typically, we do not notice micro-expressions, *yet some individuals in the general population are experts at recognizing and interpreting micro-expressions*. **A central component of Physical Intelligence is the ability to be minutely aware of small changes in your movements.** In the current study, we are testing both your "**Physical Intelligence**" as measured in the task you just performed and your micro-expressions (recorded from the camera and analyzed by specialists). Previous studies have found that persons with high Physical Intelligence have good interpersonal skills, higher capacity to show empathy and have a higher IQ.

<u>Current study:</u> The current study's goal is to detect individuals with especially high Physical Intelligence, and examine the relation between Physical Intelligence (measured in the task you just practiced) and their micro-expressions of fear and anxiety (analyzed from your facial expressions).

<u>IMPORTANT:</u> If you are found to have high physical intelligence, we will contact you and you may be eligible to participate in a series of special, innovative studies examining neurocognitive functioning in specially gifted individuals. Importantly, participation in these future studies is heavily reimbursed! "

Supplemental Material Section C: Tables of Summary Statistics for VH task

Table S1. Summary statistics of the VH task across the four experiments, Mean (S.E.M).

Category	M0	M1	M2	M3	Significant comparisons
Exp. 1: SoA rating	1.29 (0.31)	1.14 (0.28)	0.85 (0.24)	0.5 (0.31)	M0>M2 *;
Exp. 1: body ownership rating	1.135 (0.31)	0.74 (0.31)	0.48 (0.3)	0.13 (0.29)	M0>M2,M3 *
Exp.2: Temporal	0.75 (0.05)	0.44 (0.05)	0.19 (0.55)	0.11 (0.051)	M0>M1, M2, M3 ***; M1>M2, M3 ***
Exp. 2: Spatial	0.75 (0.05)	0.26 (0.06)	0.08 (0.04)	0.06 (0.04)	M0>M1, M2, M3 ***; M1>M2, M3 ***
Exp. 3: Temporal	0.87 (0.03)	0.63 (0.04)	0.30 (0.04)	0.15 (0.04)	M0>M1, M2, M3 ***; M1>M2, M3 ***; M2>M3 ***
Exp. 4: Neutral	0.86 (0.03)	0.66 (0.05)	0.28 (0.05)	0.08 (0.03)	M0>M1 **; M0>M2, M3 ***; M1>M2, M3 ***; M2>M3 ***
Exp. 4: Stress	0.86 (0.03)	0.61 (0.06)	0.27 (0.06)	0.13 (0.04)	M0>M1 **; M0>M2, M3 ***; M1>M2, M3 ***; M2>M3 ***

In experiment 1, ratings on a scale of -3 to 3 were made for subjective SoA and body ownership. In experiments 2–4, porportion of "same" responses made to agency question. Columns starting with M signify the magnitude of alteration, with M0 signifying no alteration. The right-most column reports post-hoc comparisons across the different levels of magnitude that were significant after the Bonferroni correction. Notes: $^{*}p < 0.05$; $^{**}p < 0.01$; $^{***}p < 0.001$.

Table S2. Summary statistics of the confidence rating of correct responses in the VH task across the experiments 2–4, Mean (S.E.M).

Category	M0	M1	M2	3	Significant comparisons
Exp. 2: Temporal	5.27 (0.19)	4.97 (0.27)	5.85 (0.26)	6.33 (0.23)	M0<M2**; M0<M3***; M1<M3*; M2<M3*
Exp. 2: Spatial	5.27 (0.19)	5.50 (0.25)	6.34 (0.17)	6.71 (0.08)	M0<M2, M3***; M1<M2, M3***; M2<M3*
Exp.3: Temporal	6.01 (0.15)	4.96 (0.22)	5.50 (0.21)	6.02 (0.21)	M0>M1***; M1<M2*; M1, M2<M3***
Exp. 4: Neutral	6.14 (0.15)	5.04 (0.25)	5.69 (0.17)	6.21 (0.17)	M0>M1*; M1<M2*; M1, M2<M3**
Exp. 4: Stress	6.14 (0.17)	5.19 (0.19)	5.86 (0.19)	6.26 (0.15)	M0>M1*; M1<M2**; M1 <M3**; M2<M3*

Columns starting with M signify the magnitude of alteration, with M0 signifying no alteration. The right-most column reports post-hoc comparisons across the different levels of magnitude that were significant after the Bonferroni correction. Notes: $^{*}p < 0.05$; $^{**}p < 0.01$; $^{***}p < 0.001$.
